# A Case of Inflammatory Myofibroblastic Tumor in the Abdominal Wall with Anaplastic Lymphoma Kinase and Whole Exome Sequencing Analysis

**DOI:** 10.70352/scrj.cr.25-0181

**Published:** 2025-07-16

**Authors:** Yuya Takahata, Shoichi Hazama, Toshiyuki Fujii, Masahiro Kitahara, Keisuke Hino, Kiwamu Okita, Hiroaki Nagano, Ryouichi Tsunedomi, Hiroshi Hashiyada, Kembu Nakamoto

**Affiliations:** 1 Department of Surgery, Gastroenterological Center, Shunan Memorial Hospital, Kudamatsu, Yamaguchi, Japan; 2 Department of Internal Medicine, Gastroenterological Center, Shunan Memorial Hospital, Kudamatsu, Yamaguchi, Japan; 3 Department of Gastroenterological, Breast and Endocrine Surgery, Yamaguchi University Graduate School of Medicine, Ube, Yamaguchi, Japan

**Keywords:** inflammatory myofibroblastic tumor, anaplastic lymphoma kinase, whole exome sequencing, gene mutation

## Abstract

**INTRODUCTION:**

Inflammatory myofibroblastic tumors (IMTs) are rare mesenchymal neoplasms characterized by spindle cell proliferation and inflammatory infiltration, but with an unclear etiology. Although IMTs most commonly arise in the lungs, extrapulmonary cases have been documented at various anatomical sites. Approximately 50% of IMTs harbor anaplastic lymphoma kinase (ALK) rearrangements; however, the genetic landscape of ALK-negative cases remains largely unknown. We report a rapidly growing IMT in the right rectus abdominis muscle and present whole-exome sequencing (WES) findings that revealed novel genetic mutations beyond ALK rearrangements.

**CASE PRESENTATION:**

A 38-year-old woman with no significant medical history presented with a rapidly enlarging mass in the right lower abdomen. Computed tomography showed a well-defined tumor on the dorsal side of the right rectus abdominis muscle exhibiting progressive enhancement. Fine-needle biopsy initially suggested the presence of proliferative fasciitis. Owing to rapid tumor growth from 40 to 61 mm within 3 months, laparoscopic surgical resection was performed, including a portion of the posterior sheath and rectus abdominis muscle. Pathological examination confirmed the presence of an IMT and revealed spindle cell proliferation, nuclear atypia, and inflammatory infiltration. Immunohistochemical analysis revealed positivity for smooth muscle actin (SMA) and ALK, partial positivity for desmin, and negativity for cluster of differentiation 34 (CD34) and cytokeratin, compatible with an IMT. WES identified 7 genetic mutations, none of which have been previously reported for IMT in the catalogue of somatic mutations in cancer (COSMIC) database, suggesting novel genetic associations.

**CONCLUSIONS:**

This case highlights a rare and rapidly growing IMT in the rectus abdominis muscle and underscores the value of molecular analysis in understanding the pathogenesis of IMT. Identification of novel mutations through WES expands the genetic landscape of IMT and may provide insights into tumorigenesis and potential therapeutic targets. Further research is required to explore the clinical implications of these mutations in IMT progression and treatment.

## Abbreviations

ALK
: anaplastic lymphoma kinase
CAM
: cytokeratin antibody
CD34
: cluster of differentiation 34
CFHR2
: complement factor H related 2
CK
: cytokeratin
COSMIC
: catalogue of somatic mutations in cancer
EGFR
: epidermal growth factor receptor
EPHB1
: ephrin type-B receptor 1
EPS15
: epidermal growth factor receptor pathway substrate 15
IHC
: immunohistochemistry
IL15
: interleukin15
IMT
: inflammatory myofibroblastic tumor
NGS
: next generation sequencing
PF
: proliferative fasciitis
PRKAB1
: protein kinase 5’ adenosine monophosphate activated non-catalytic subunit beta 1
SMA
: smooth muscle actin
SMARCC2
: switch/sucrose non-fermentable-related, matrix-associated, actin-dependent regulator of chromatin, subfamily C member 2
SWI/SNF
: switch/sucrose-non-fermentable
TENM1
: teneurin transmembrane protein
TMEM233
: transmembrane protein 233
WES
: whole-exome sequencing

## INTRODUCTION

IMTs are distinctive mesenchymal neoplasms characterized by spindle cell proliferation with inflammatory infiltrates.^1)^ IMTs are very rare, occurring in less than 1 in 1 million people. It is estimated that 150–200 people are diagnosed with IMTs annually in the United States of America (https://www.cancer.gov/pediatric-adult-rare-tumor/rare-tumors/rare-soft-tissue-tumors/inflammatory-myofibroblastic-tumor). Most IMTs occur in the first 3 decades of life (mean age = 10 years), although cases can also occur throughout adulthood. IMTs can occur throughout the body, but most frequently involve the lungs, abdomen, pelvis, and retroperitoneum. The symptoms depend on the site of involvement.^2)^ Local recurrence may occur after initial surgery, with a low risk of distant metastasis. IMTs are soft tissue tumors with intermediate biological potential, with a small fraction behaving aggressively.^3)^

Although the etiology of IMTs remains unclear, recent molecular studies have revealed that approximately 50% of IMT cases harbor rearrangements in the ALK gene.^4)^ Molecular abnormalities in ALK-negative IMT cases remain largely unknown. Although there have been many reports on ALK mutations, there have been very few reports on the analysis of genetic mutations other than ALK mutations using WES.

Here, we report a case of IMT that rapidly developed in the patient’s right rectus abdominis muscle. Given the patient’s relatively advanced age at onset compared with previously reported cases, the tumor's aggressive growth, and the limited genomic data available for IMTs beyond ALK alterations, we performed WES to investigate potential novel genetic drivers of tumor progression.

## CASE PRESENTATION

**Fig. 1:**
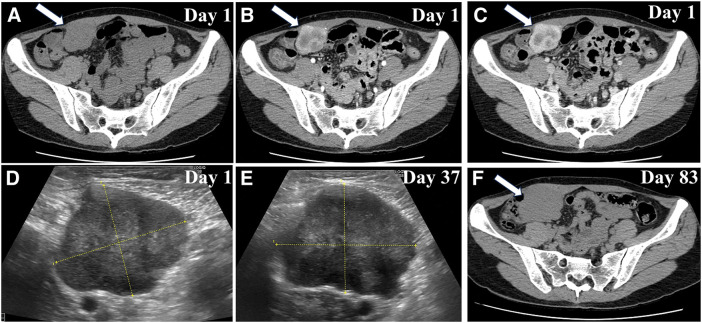
Imaging findings (**A**) Contrast enhanced CT on February 7. A 40 × 35 mm mass was identified in contact with the right lower abdomen. (**B** and **C**) Early enhancement was observed at the margins, with delayed enhancement within the interior. (**D**) Abdominal US on February 7. A hypoechoic mass (41.8 × 35.2 mm) was observed in the right lower abdomen. The mass had a lobulated shape, with a mix of hyper- and hypoechoic areas internally. (**E**) Abdominal US findings on March 15 revealed that the mass had increased in size by 9 mm in the long axis and 7 mm in the short axis over 1 month. (**F**) Plain CT on April 30, showing that the mass had increased to 61.3 × 53.1 mm. US, Ultrasonography

**Fig. 2:**
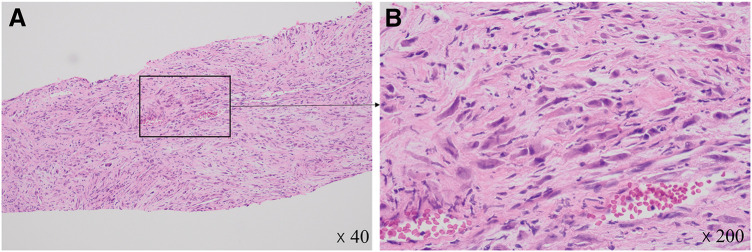
Preoperative pathological findings (**A**) Low power field of HE staining. (**B**) High power field of HE staining revealed spindle-shaped nuclei, with fibroblast-like cellular components proliferating in fascicular or intricate patterns. Within these structures, occasional ganglion-like giant cells were observed. HE, hematoxylin and eosin

**Fig. 3:**
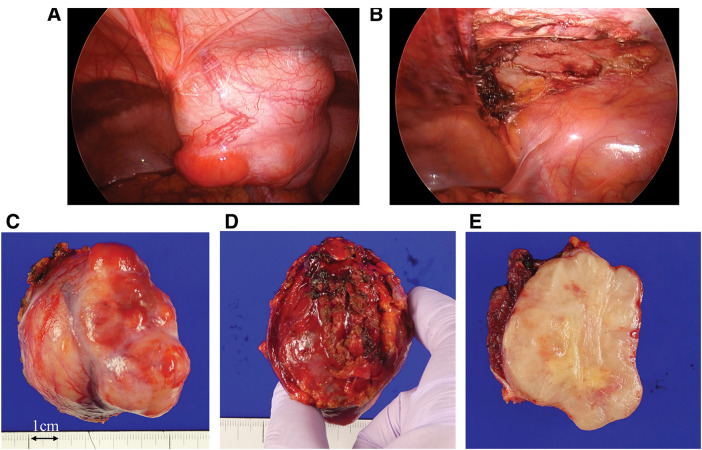
Intraoperative findings and postoperative specimens (**A**) A well-defined tumor was observed on the lateral aspect of the right lower abdominal wall, adjacent to the inferior epigastric artery and vein. (**B**) The tumor was completely resected with partial excision of the peritoneum and muscle. (**C**) Peritoneal side of the specimen, (**D**) abdominal wall side of the specimen, and (**E**) sectional view of the specimen.

A 38-year-old woman with no significant medical history presented with a right-sided lower abdominal mass. No special findings were found in the blood tests. CT revealed a well-defined localized tumor on the dorsal side of the right rectus abdominis muscle (**Fig. 1A**–**1C**). Early enhancement was observed around the tumor (**Fig. 1B**) with increased enhancement in the late phase (**Fig. 1C**). Fine-needle biopsy indicated that spindle-shaped fibroblasts proliferated in a bundled and convoluted pattern. Nuclear atypia was mild, suggesting PF, which differed from the postoperative pathological diagnosis (**Fig. 2A** and **2B**). Due to rapid growth in size from 40 to 61 mm within 3 months (**Fig. 1E** and **1F**), surgical intervention was planned, and a laparoscopic procedure was performed to completely remove the tumor, with resection of a portion of the posterior sheath of the rectus abdominis and part of the rectus abdominis muscle, ensuring adequate margins (**Fig. 3A** and **3B**). The tumor was 64 mm long and 58 mm short, with clear borders, well-defined margins, elastic hardness, and fullness (**Fig. 3C** and **3D**). The tumor cross section was white, firm in consistency, and homogeneous in nature (**Fig. 3E**).

**Fig. 4:**
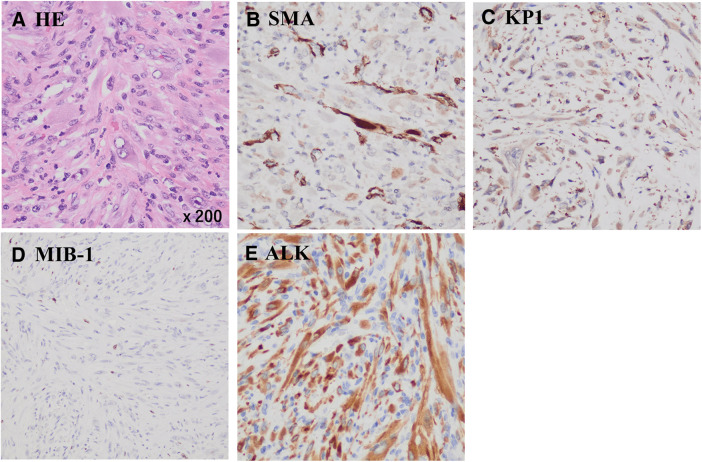
Postoperative pathological findings (**A**) HE stain findings revealed spindle-shaped nuclei and eosinophilic cytoplasm, proliferating in a trabecular and convoluted pattern, with infiltration of inflammatory cells, such as lymphocytes. (**B**) IHC was positive for SMA and (**C**) KP-1, with an (**D**) MIB-1 index <10%. (**E**) IHC was positive for ALK. ALK, anaplastic lymphoma kinase; HE, hematoxylin and eosin; IHC, immunohistochemistry; SMA, smooth muscle actin

Postoperative pathological examination revealed that tumor cells had spindle-shaped nuclei, eosinophilic cytoplasm, marked nuclear atypia, and mitotic figures. The tumor cells proliferated in a trabecular and convoluted pattern and were infiltrated by inflammatory cells, such as lymphocytes (**Fig. 4A**). Tumor cells with significant nuclear atypia were not evident in the preoperative specimen, leading to the diagnosis of PF. However, the postoperative specimen revealed proliferation of tumor cells with marked nuclear atypia, supporting the diagnosis of IMT.

Other differential diagnoses included inflammatory leiomyosarcoma and sarcomatoid mesothelioma. Therefore, IHC was performed to distinguish between these entities. In this case, immunohistochemical staining was positive for SMA (**Fig. 4B**), partially positive for desmin, and positive for KP-1 (**Fig. 4C**), but negative for CD34, CK (AE1/AE3), and CK (CAM5.2), with an MIB-1 index of <10% (**Fig. 4D**) and ALK positivity (**Fig. 4E**), confirming the diagnosis of IMT (**Table 1**).

**Table 1:** Differential diagnoses according to immunohistochemical findings

	SMA	Desmin	KP-1	CD34	MIB-1	CK (AE1/3)	CAM5.2	ALK
This case	+	Partially +	+	–	<10%	–	–	+
Proliferative fasciitis (PF)	+	+ ~ –	+	–	Low	–	–	–
		(+ < –)	(Low)					
Inflammatory myofibroblastic tumor (IMT)	+	+ ~ –	+	–	Low	–	–	+ ~ –
			(High)					
Inflammatory leiomyosarcoma	+	+	–	–	High	–	–	–
Sarcomatoid mesothelioma	+ ~ –	–	+	–	High	+	+	–
	(+ < –)							

ALK, anaplastic lymphoma kinase; CAM, cytokeratin antibody; CD34, cluster of differentiation 34; CK, cytokeratin; SMA, smooth muscle actin

WES was performed using DNA from the peripheral blood and tumor, as previously described.^5)^ In brief, 3 μg of DNA from each sample was used to prepare *in vitro* DNA libraries using the Sure Select Target Enrichment System (Agilent Technologies Japan, Tokyo, Japan) with the Sure Select XT Reagent Kit (Agilent Technologies Japan) and the Sure Select Human ALL Exon V5 + UTRs (Agilent Technologies Japan), producing a total target size of 75 Mb. Paired-end fragments (100 bp × 2) were sequenced on an Illumina HiSeq 2000 sequencing platform (Illumina, San Diego, CA, USA) at the Dragon Genomics Center (TaKaRa Bio, Mie, Japan).

WES identified 7 genes with a mutation frequency of >10% in the tumor (**Table 1**). These genes were searched using COSMIC (https://cancer.sanger.ac.uk/cosmic), a resource widely used to identify targets for cancer research and treatment. One genetic mutation, EPHB1, has been reported in a case of malignant melanoma; however, the other genetic mutations have not been previously reported in COSMIC and thus represent novel findings (**Table 2**). Although there have been no reports of gene mutations at the same position, there have been some reports of mutations in the same protein. Multiple mutations involved in tumor formation and progression were identified, which were likely related to tumor formation, progression, and immune evasion (**Table 3**).

**Table 2:** Genes with mutations in the tumor

Genes with mutation (frequency >10%)	Previous report	Freq. (%)
	on COSMIC	
EPS15|c.181G>A|p.Asp61Asn|	No matching records found	11.5
CFHR2|c.707T>C|p.Ile236Thr|	No matching records found	12.0
EPHB1|c.1724A>T|p.Tyr575Phe|	Malignant melanoma	14.0
IL15|c.444G>T|p.Leu148Phe|	No matching records found	11.5
SMARCC2|c.1051A>C|p.Asn351His|	No matching records found	12.8
TMEM233|c.268A>G|p.Ile90Val|	No matching records found	13.0
TENM1|c.3878G>T|p.Cys1293Phe|	No matching records found	13.7

CFHR2, complement factor H-related 2; COSMIC, catalogue of somatic mutations in cancer; EPHB1, Ephrin type-B receptor 1; EPS15, epidermal growth factor receptor pathway substrate 15; IL15, interleukin15; SMARCC2, SWI/SNF-related, matrix-associated, actin-dependent regulator of chromatin, subfamily C member 2; SWI/SNF, switch/sucrose-non-fermentable; TENM1, teneurin transmembrane protein; TMEM233, transmembrane protein 233

**Table 3:** Proteins with gene mutations and their roles

Mutated protein	Main role under normal circumstances	Relationship with disease
EPS15	Related to the epidermal growth factor signaling pathway	Lung cancer, breast cancer
CFHR2	Plays a role in the immune response and inflammation control	Complement-mediated kidney disease
EPHB1	Role as a tumor suppressor	Bladder cancer, glioma
IL15	Promotes natural immunity (activation of NK cells and CD8+ T cells)	Leukemia-derived and solid tumor
SMARCC2	Involved in transcription regulation and DNA repair. Contributes to cell cycle and differentiation	Some types of cancers
TMEM233	Involved in signal transduction and cell homeostasis	–
TENM1	Involved in neurogenesis, synapse formation, and cell–cell adhesion	Thyroid carcinoma, pituitary tumor, and glioblastoma

CFHR2, complement factor H-related 2; EPHB1, Ephrin type-B receptor 1; EPS15, epidermal growth factor receptor pathway substrate 15; IL15, interleukin 15; SMARCC2, SWI/SNF-related, matrix-associated, actin-dependent regulator of chromatin, subfamily C member 2; SWI/SNF, switch/sucrose-non-fermentable; TENM1, teneurin transmembrane protein; TMEM233, transmembrane protein 233

These analyses were conducted in accordance with the Declaration of Helsinki and approved by the Institutional Ethics Review Boards of Shunan Memorial Hospital (R04-08) and Yamaguchi University (Approval No. H17-83). Written informed consent was obtained from the patient for the publication of this report and the accompanying analyses.

## DISCUSSION

IMTs are distinctive mesenchymal neoplasms characterized by spindle cell proliferation with inflammatory infiltrates.^1)^ In this case, postoperative pathology revealed spindle-shaped tumor cells with infiltrating inflammatory cells, consistent with IMT. This patient was 38 years old at onset, whereas previous reports have indicated that the onset is more common in individuals in their 20s or younger,^6)^ making this case unusual in terms of age. Although the lungs are the most common site of IMT, a report summarizing 84 cases of extrapulmonary IMTs found that 64 involved the abdomen, retroperitoneum, or pelvis, indicating that abdominal wall involvement, as seen in this case, is relatively common in IMT.^7)^ The growth rate of IMTs varies widely among reported cases. Some lesions have been observed to regress spontaneously,^8)^ while others demonstrate slow or moderate progression. Conversely, there is also a case report of an IMT that rapidly enlarged from 13 to 82 mm within 2 months.^9)^ These findings suggest that IMTs do not follow a consistent growth pattern. In the present case, the tumor increased in size from 40 to 61 mm over a 3-month period, which is a relatively rapid growth rate, yet still within the range previously reported. Complete surgical resection remains the primary treatment modality for IMTs.^3)^ In this case, the tumor originated on the dorsal side of the right rectus abdominis muscle. Preoperative imaging (ultrasound and CT) revealed a well-defined, localized lesion with no evidence of infiltration into surrounding structures. Based on these findings, we determined that the tumor could be completely resected via a laparoscopic approach, which was successfully achieved. However, we recognize that in previously reported cases where the tumor invaded adjacent muscle layers extensively, open resection with or without abdominal wall reconstruction was required to achieve negative surgical margins.^10,11)^ Had the tumor in our case progressed further, open surgery might also have been necessary. While minimally invasive techniques such as thoracoscopic or laparoscopic resection are frequently employed for IMTs of the lung and intra-abdominal organs,^12,13)^ their use in abdominal wall IMTs remains rare. This case suggests that, under suitable conditions, laparoscopic surgery can be a viable and effective option even for IMTs located in the abdominal wall. No recurrence was observed 1 year postoperatively; however, numerous reports indicate that local recurrence is common after initial surgery, necessitating careful follow-up.^3)^

Studies have reported that approximately 50% of IMTs have alterations in the ALK gene located on chromosome 2p23.^4)^ ALK IHC is the most useful marker because the reactivity of neoplastic spindle cells with ALK corresponds strongly to the presence of a clonal ALK rearrangement.^14)^ In this case, ALK positivity on IHC confirmed the presence of an ALK rearrangement, which was the decisive factor in the diagnosis of IMT.

NGS, a recently developed automated sequencing method, enables the analysis of large fragments of DNA and RNA isolated from formalin-fixed paraffin-embedded tissues and paired fresh-frozen samples.^15)^ NGS allows detection of the sequence of nitrogenous bases in nucleic acids, and thus, detects even the smallest molecular genetic changes, such as point mutations, insertions, and deletions.^16)^ The application of NGS may improve our understanding of the genetic etiopathology of IMTs.^17)^ In this case, WES was performed to identify genetic mutations other than ALK, which may help in understanding the pathophysiology of IMTs. As a result, we identified more than 10% of genetic mutations in EPS15, CFHR2, EPHB1, IL15, SMARCC2, TMEM233, and TENM1 (**Table 1**). A search of COSMIC (https://cancer.sanger.ac.uk/cosmic) in 2025 revealed that this was the first reported case of genetic mutations in IMT. EPS15 is primarily involved in endocytosis and the regulation of EGFR and other signaling pathways, as well as being important for cell proliferation and differentiation. Mutations in EPS15 have been shown to be associated with cancers with abnormalities in the EGFR pathway. As a crucial player in terminating growth factor signaling, EPS15 plays an important role in many malignancies, including breast cancer.^18)^ CFHR2 is involved in regulation of the complement cascade and is responsible for the immune response and inflammatory control. In particular, CFHR2 is functionally related to the complement regulatory protein complement factor H. CFHR2 gene mutations are associated with complement pathway abnormalities and may be associated with complement-mediated renal diseases.^19)^ EPHB1 is an ephrin receptor that contributes to cell–cell adhesion, cell migration, and morphogenesis and is involved in developmental processes and inhibition of tumor invasion. It acts as a tumor suppressor, and loss of function due to mutations is associated with accelerated cancer progression. Overexpression or downregulation of certain EPH receptors has been shown to be associated with tumorigenesis in some types of cancers.^20)^ IL15 is a cytokine that promotes the activation of NK and CD8+ T cells, which play a central role in regulating the immune system. The expression of IL-15 and/or IL-15 receptors can be detected in several leukemia- and solid tumor-derived cell lines, which display pro-tumorigenic properties.^21)^ SMARCC2 is a component of the SWI/SNF chromatin remodeling complex and is involved in transcriptional regulation and DNA repair, contributing to cell cycle and differentiation.^22)^ SMARC dysregulation in cancer can lead to either loss-of-function of tumor suppressors and/or gain-of-function oncogenic mechanisms.^23)^ TMEM233 is a transmembrane protein that localizes in the plasma membrane and may be involved in signal transduction and cellular homeostasis. TMEM233/PRKAB1 fusion is a critical driver and may serve as a therapeutic target for Hurthle cell carcinoma.^24)^ TENM1 is involved in neurogenesis, synapse formation, and cell–cell adhesion.^25)^ TENM1 dysregulation is associated with several types of tumors. However, data on the association between TENM1 deregulation and tumor progression are scarce and confined to a few tumor types, such as thyroid carcinoma, pituitary tumors, and glioblastoma. WES in this case revealed mutations in several genes, including EPS15, CFHR2, EPHB1, IL15, and SMARCC2, which are involved in tumor-related processes such as cell signaling,^18)^ immune regulation,^21)^ chromatin remodeling, and tumor suppression.^22)^ Notably, IL15 and CFHR2 may contribute to immune evasion within the tumor microenvironment.^19,21)^ We also found mutations in TMEM233 and TENM1, genes not previously associated with IMT. TMEM233 encodes a membrane protein and may play a role in cell signaling and homeostasis, and a gene fusion involving TMEM233 has been reported in Hurthle cell carcinoma.^24)^ TENM1 is involved in nerve development and cell adhesion and has been implicated in thyroid and brain cancers.^26)^ While the roles of these genes in IMT remain unclear, they may be related to tumor architecture or growth. These findings may help us understand IMT better and suggest that immune dysregulation, aberrant signaling, and structural genetic alterations may be involved in IMT pathogenesis.

In this case, WES was performed postoperatively and did not influence surgical planning or margin assessment. Given the diagnostic uncertainty of IMT prior to resection, the current utility of WES in preoperative decision-making remains limited. However, molecular profiling may help in stratifying recurrence risk or identifying candidates for adjuvant therapy in selected cases. Immunologically relevant mutations, such as those in IL15 or CFHR2, suggest potential for immune-based monitoring or future targeted therapies. These findings may also support postoperative referral to oncology or genetics services.

A notable limitation of this report is the lack of functional validation for the detected mutations. Although this is a single-case study, further research using transcriptomic or proteomic analyses is needed to clarify the biological relevance of these genetic alterations and to determine their potential role in guiding treatment strategies.

## CONCLUSIONS

This case highlights a rare and rapidly growing IMT in the rectus abdominis muscle and underscores the value of molecular analysis in understanding IMT pathogenesis. Identification of novel mutations through WES expands the genetic landscape of IMTs, which may provide insights into tumorigenesis and potential therapeutic targets. Further research is required to explore the clinical implications of these mutations in IMT progression and treatment.

## ACKNOWLEDGMENTS

The authors thank Dr. Tokuhiro Ishihara, Dr. Toshiaki Kamei, and Mr. Yosuke Nagahiro for their pathological analysis of this work. We would like to thank Editage (https://asco.editage.com/services/) for English language editing.

## DECLARATIONS

### Funding

The authors received no specific funding for this work.

### Authors’ contributions

YT, SH, TF, and MK treated the patient.

YT and SH collected the clinical data and wrote the manuscript.

YT, SH, RT, HN, HH, and KN applied and assessed the results of the genetic examination and discussed them.

YT, SH, KH, and KO applied and assessed the results of the immunohistochemical examination and discussed them.

All authors attended the discussion and have read and approved the final manuscript.

### Availability of data and materials

The data that support the findings of this study are available from the corresponding author upon reasonable request.

### Ethics approval and consent to participate

This report has been performed in accordance with the Declaration of Helsinki and approved by the Institutional Ethics Review Boards of Shunan Memorial Hospital (R04-08) and Yamaguchi University (Approval No. H17-83). Written informed consent was obtained from the patient for the accompanying analyses.

### Consent for publication

Written informed consent was obtained from the patient for publication of this case report.

### Competing interests

The authors declare no competing interests with respect to this report.
